# Survival during the Breeding Season: Nest Stage, Parental Sex, and Season Advancement Affect Reed Warbler Survival

**DOI:** 10.1371/journal.pone.0148063

**Published:** 2016-03-02

**Authors:** Kaja Wierucka, Lucyna Halupka, Ewelina Klimczuk, Hanna Sztwiertnia

**Affiliations:** 1 Department of Biological Sciences, Macquarie University, Sydney, Australia; 2 Université Paris-Saclay, Université Paris-Sud, CNRS, UMR 9197, Institut des Neurosciences Paris-Saclay, Orsay, France; 3 Ornithological Station, University of Wroclaw, Wroclaw, Poland; 4 Museum of Natural History, Faculty of Biology, University of Wroclaw, Wroclaw, Poland; CSIC-EEZA, SPAIN

## Abstract

Avian annual survival has received much attention, yet little is known about seasonal patterns in survival, especially of migratory passerines. In order to evaluate survival rates and timing of mortality within the breeding season of adult reed warblers (*Acrocephalus scirpaceus*), mark-recapture data were collected in southwest Poland, between 2006 and 2012. A total of 612 individuals (304 females and 308 males) were monitored throughout the entire breeding season, and their capture-recapture histories were used to model survival rates. Males showed higher survival during the breeding season (0.985, 95% CI: 0.941–0.996) than females (0.869, 95% CI: 0.727–0.937). Survival rates of females declined with the progression of the breeding season (from May to August), while males showed constant survival during this period. We also found a clear pattern within the female (but not male) nesting cycle: survival was significantly lower during the laying, incubation, and nestling periods (0.934, 95% CI: 0.898–0.958), when birds spent much time on the nest, compared to the nest building and fledgling periods (1.000, 95% CI: 1.00–1.000), when we did not record any female mortality. These data (coupled with some direct evidence, like bird corpses or blood remains found next to/on the nest) may suggest that the main cause of adult mortality was on-nest predation. The calculated survival rates for both sexes during the breeding season were high compared to annual rates reported for this species, suggesting that a majority of mortality occurs at other times of the year, during migration or wintering. These results have implications for understanding survival variation within the reproductive period as well as general trends of avian mortality.

## Introduction

Survival, along with its variations in patterns and probability over time, makes up one of the most important elements in understanding the life-history dynamics of a species [1]. This is especially relevant for short-lived but iteroparous animals [2] such as passerines. Adult survival over time is a crucial demographic parameter as it can affect population size and influence the decline of many bird species [3, 4]. However, an overwhelming majority of research has evaluated only annual survival rates (e.g. [5, 6, 7]) and these studies do not give insight into fine-scale timing of mortality.

The number of studies investigating survival within shorter parts of an annual cycle has been increasing in recent years (e.g. [8, 9, 10]), but the detected patterns differ enormously between species. The highest mortality may occur at wintering grounds [11], during migration [12, 13], or the breeding season [14, 15, 16]. Differences are present even within the same ecological groups, e.g. sedentary woodland species from temperate zones: willow tits and black-capped chickadees showed lowest survival in early winter [2, 17], nuthatches were at risk in late winter and early spring [18], and blackbirds showed highest mortality during the breeding season [15]. Information about within-season survival is especially scarce for migratory passerines and data are restricted to only a few species [2, 13, 15, 19, 20, 21, 22].

Bird survival may vary by sex (e.g. [19, 20]). We expect to find lower survival in the sex taking on more parental duties, as higher reproductive effort and increased predation risk are a function of time spent on the nest [23, 24]. Higher reproductive effort can also result in increased susceptibility to parasitism or decreased immune system functioning leading to fitness costs and therefore negatively influencing parental survival [25, 26, 27]. Female mortality tends to be higher than that of males in passerines [28, 29] and several studies provided evidence for low survival of females during the breeding season [19, 21, 22, 30, 31]. However, in other species (including some with female-biased parental care) survival of both sexes was similar during the reproductive season [18, 2, 13, 15] or even higher for females than males [20].

Mortality during the breeding season may sometimes be associated with human activity, like mowing [21] or subsistence harvest on the breeding grounds [12], but natural factors are usually the main cause of death. Previous research provided evidence for predation being an important cause of bird mortality during the breeding season. The studies revealed that parental birds were killed on or near the nest (e.g. [9, 19, 30, 32]). If on-nest predation is the main factor responsible for adult mortality we should expect to find lower survival rates in periods of the season when most birds incubate. Decreasing survival during the breeding season may be related to accumulating energetic costs [25, 26, 27]. However, increased predation in later parts of the breeding season, or higher levels of parental nest defense exposing birds to a higher risk of injury or death would result in similar trends [27, 33]. Furthermore, seasonally declining survival observed in a population could be also a result of an increase in the proportion of naive breeders over the reproductive season.

We are not aware of any research investigating differences in parental survival between the various stages of a nesting cycle (laying, incubation, feeding of the nestlings, or fledglings) in altricial birds. However, several studies reported high mortality of incubating parents, especially females [21, 22, 30, 31, 34]. Recently, Blomberg et al. [16] showed that successful reproduction was related to reduced survival after the breeding season (though lower survival rates were found during nesting).

In this paper we analyse survival rates of adult Eurasian reed warblers *Acrocephalus scirpaceus* during the breeding season. In particular, we test whether females have lower survival rates than males, and if survival rates change over the breeding season. We are also the first to demonstrate that survival differs between various stages of the nesting cycle.

## Materials and Methods

### Study species

The Eurasian reed warbler is a small passerine, with a long reproductive season (May-August), breeding mostly in Palearctic reedbeds. The species is a long-distance migrant wintering in sub-Saharan Africa [35] and the arrival of both sexes to the breeding ground lasts for more than a month (in our population from ca. 25^th^ April to 15^th^ June). Reed warblers reach very high breeding densities (that may exceed 200 pairs per 10 ha), and hence hold small territories and move short distances within a breeding season [35]. They suffer high nest losses (55.1% on average in Europe; [36]) and frequently re-nest, laying up to five clutches per season. Although the species is predominantly monogamous, polygamy has been recorded in some west-European populations [37, 38], but has never been observed in our study site [39]. Pairs generally remain together throughout the whole breeding season. Furthermore, both parents are engaged in incubation and feeding of the young, although females spend more times on the nest compared to males [35, 40].

### Study population and data collection

The data were collected between 2006 and 2012 in the Stawy Milickie (Milicz fish-ponds) nature reserve (SW Poland) on a 4 ha study plot located on the "Słoneczny Górny" pond (center of the study plot: 51.5385°N, 17.3390°E). Each year the study site was monitored from the time of arrival of the first individuals (late April/early May) until the fledging of the last young (mid-August). Due to long-lasting arrival from wintering grounds and high nest losses, breeding was highly asynchronous in our breeding population [33, 38]. This enabled us to analyse the effect of season progression and nesting stage on survival patterns. Birds were mist-netted and individually marked (combinations of three coloured and one metal ring) throughout the breeding season. A majority of nests were discovered during the building stage by observing nest-building and mate-guarding behaviour. Parental birds were usually recorded 6–7 times at their nests/in territories: 1) while observing singing males in their territories (male mapping) and pairs building their nests (nest searching), 2) during observations of mate-guarding behaviour corresponding with the nest-building period (most pairs were observed continuously for about two hours before laying), and 3) by video-recording (JVC Everio GZ330) all available nests on three occasions for at least two hours: on the day of laying of the second egg, as well as during the incubation and nestling stage (on the eighth day after hatching). When possible, we also recorded parents feeding the fledglings. Additionally, the presence of birds on the study plot was recorded during mist-netting. Nests were visited usually every two days, and daily during egg-laying and before the expected hatching and fledging dates. Following nest loss, the character of disturbance was noted and the area around the nest was searched for any remains of chicks or adults. A pair would usually start building a new nest soon after the first/previous nest failure close to the previous nest [33] (generally on the same day). We made every effort to find successive nests of breeding pairs as soon as possible, and hence were able to monitor most birds throughout the whole breeding season. Sampling effort (frequency of nest visits, number of nest recordings etc.) was kept constant throughout the breeding season. Frequent observations and/or recordings enabled us to accurately assess the day of disappearance of a parent. For other details concerning study procedures see Halupka et al. [33, 38, 41].

As reed warblers are highly philopatric within a breeding season, distances between two successive nests of a pair are very short: on average 13 and 14 m after nest success and nest failure, respectively [33]. All birds taken into analysis remained at the study site after nest failure, making it possible to monitor them throughout the whole breeding season and eliminating the problem of dispersal after nest failure. We assumed the death of an individual when: 1) a corpse or remains were found by the nest or within the study plot; 2) an animal was not observed at the nest within a 2–4 hour period of standard nest observation, and never again afterwards. We carefully examined each case of parental disappearance from active nests (containing offspring), and none of these individuals were recorded later in the focal breeding season or in any of the following seasons. These birds were also not observed on the neighbouring plot, where our colleague conducted another reed warbler project in 2007–2010. We never observed the disappearance of the studied birds from their territories between breeding attempts (e.g. after breeding failure). In contrast, all cases of assumed mortality (when birds were found dead or disappeared) occurred at nests containing offspring. In theory, we cannot exclude the possibility that birds disappearing from active nests dispersed (and not died), however in this case a bird would have to abandon a brood, separate from its partner, leave the study area and never return. Considering the biology of the species this is extremely unlikely. Brood desertion has never been recorded in the species (see [38] for the review of studies involving colour ringing). Furthermore, in this monogamous species, both parents are needed to successfully raise young [37, 42]. Consequences of mate removal in the species were demonstrated experimentally by Duckworth [42]: widowed males did not continue incubation or brooding after female disappearance but abandoned their clutches and started to behave as bachelors: they resumed singing and attempted to attract new mates, while widowed females frequently had lower breeding success (starving of the young also occurred). After each parental disappearance we observed the same behaviours, as recorded by Duckworth [42], except for five cases when the nestlings were also killed during the predation event.

### Ethics statement

The study involved mist-netting and ringing adult reed warblers, as well as frequent nest visits. All procedures regarding this field study were conducted according to the respective legislation in Poland.

The reed warbler is a common breeding species with a status of least concern. However, as the study was conducted in the protected area of the nature reserve, each year we obtained field permits from the Regionalna Dyrekcja Ochrony Środowiska (Regional Directorate of Nature Protection) in Wroclaw, Poland. All observations and sampling procedures were reviewed and approved as part of obtaining the field permit. Ringing licenses were issued by the Ornithological Station of the Polish Academy of Sciences based on the decisions of Polish Ministry of Environment. This research did not involve any experimental manipulations, or the sacrifice of animals.

### Statistical analysis

We adopted nest survival models available in the program MARK, version 7.1 [43], to calculate daily survival rates (DSR) of the studied adult reed warbler population. These models can be used for analyzing survival data (of individuals) collected using ragged intervals among animals and over time [44]. All individuals used for the analyses were adults, and were sexually mature. The first sightings of individuals in a given season, the last sightings of live animals, days the deaths of individuals were discovered, as well as information whether an individual survived the breeding season, were used to generate an encounter history. Julian dates were used, with day 1 defined as the day the first animal was sighted in a breading season. The length of the breeding season was 108 days. As a number of individuals returned to the same breeding ground each year, some birds were observed during multiple years. However, for the purposes of this analysis they were considered as independent observations. We only included birds observed throughout the whole breeding season, from their settlement in territories in May-June until July-August, when they stopped breeding and started migration.

Various models were tested (Table 1). We began the model selection with model *φ years(t) days(t)* (Table 1, model 11) where DSR varied between years and days. Next, we tested a model where survival was constant throughout the breeding season and did not vary between years (Table 1, model 8). A model with DSR varying between years, but constant throughout days (Table 1, model 9) was evaluated to assess annual variation. Furthermore, we ran a model with no difference present between years, but with DSR varying between days (Table 1, model 10). We then investigated the possible influence of the progression of the breeding season on DSR values: models dividing the breeding season into 2, 3, and 4 equal intervals were tested (Table 1, models 5, 6, 7, respectively). Following that, we added the effect of sex to the different interval models–to evaluate whether survival modelled separately for females and males will better suit our data (Table 1, models 2, 3, 4). We suspected that female survival should be more affected by the advancement of the breeding season than that of males. Based on that, as well as preliminary inspections of the results of the models described above, an additional model, being a mix of best interval model and best general model was created (Table 1, model 1).

**Table 1:** Model selection for estimating survival rates of reed warblers between 2006–2012 in the "Stawy Milickie" nature reserve, Poland.

No.	Model	AICc	Δ AICc	AICc Weight	Model Likelihood	NP	Deviance
**1.**	**{φ years(.) M days(.) F 3 intervals}**	**242.841**	**0.000**	**0.641**	**1.000**	**4**	**234.840**
2.	{φ years(.) 3 intervals; sex}	244.847	2.006	0.235	0.367	6	232.844
3.	{φ years(.) 2 intervals; sex}	246.422	3.581	0.107	0.167	4	238.421
4.	{φ years(.) 4 intervals; sex}	250.816	7.975	0.012	0.019	8	234.811
5.	{φ years(.) 3 intervals}	252.797	9.956	0.004	0.007	3	246.796
6.	{φ years(.) 2 intervals}	256.836	13.995	0.001	0.001	2	252.835
7.	{φ years(.) 4 intervals}	257.202	14.361	0.000	0.001	4	249.201
8.	{φ years(.) days(.)}	260.730	17.888	0.000	0.000	1	258.729
9.	{φ years(t) days(.)}	264.178	21.337	0.000	0.000	7	250.174
10.	{φ years(.) days(t)}	436.592	193.751	0.000	0.000	108	219.731
11	{φ years(t) days(t)}	1542.272	1299.431	0.000	0.000	671	166.598

The most parsimonious model has the lowest corrected Akaike's information criterion (AICc) value. ΔAICc: difference of AICc value from the best suited model (bold); NP: number of parameters; AICc weights, model likelihood and deviance as defined by Cooch and White [44]. Model notations– φ: survival, t: time dependent, ".": constant through time; M–male, F—female.

Survival rates during different nesting stages for both males and females were evaluated. For this analysis, the same model as for the first analysis was used, however, new encounter histories were created. The time span was now not the whole breeding season, but a single nesting cycle. Consequently, the first sightings of individuals in a given nesting cycle, the last sightings of a live animals, days when the deaths of individuals were found, as well as information whether an individual survived the nesting cycle were used. First, a basic model taking into account five distinct intervals: nest building (5 days; B), laying (4 days; L), incubation (10 days; I), nestling (12 days; N), and fledgling (14 days; F) (Table 2, model 5) was calculated for males and females. Based on estimates obtained from the basic model we pooled nesting stages during which similar mortality was exhibited ("+" denotes stages being modeled as one interval) to evaluate similarities between given stages. Pooling nest stages was done in two steps: 1) pooling stages during which any mortality was observed (φ<1.00); 2) pooling stages that had a difference in survival of 0.005 or less. Several model possibilities were first explored for females (Table 2, models 3 and 4). Once the best suited model for females was obtained, models exploring different scenarios for males were created (Table 2, model 1 and 2). The survival rate was assumed to be constant within each interval.

**Table 2:** Model selection for estimating survival rates of reed warblers between 2006–2012 in the "Stawy Milickie" nature reserve, Poland in relevance to nest stage.

No.	Model	AICc	Δ AICc	AICc Weight	Model Likelihood	NP	Deviance
**1.**	**{φ F (B, L+I+N, F); M (B+L+I+N+F)}**	**231.082**	**0.000**	**0.867**	**1.000**	**4**	**223.080**
2.	{φ F (B, L+I+N, F); M (B, L, I+N, F)}	235.784	4.702	0.083	0.095	7	221.777
3.	{φ F (B, L+I+N, F); M (B, L, I, N, F)}	237.725	6.643	0.031	0.036	8	221.717
4.	{φ F (B, L+I, N, F); M (B, L, I, N, F)}	239.619	8.537	0.012	0.014	9	221.609
5.	{φ F (B, L, I, N, F); M (B, L, I, N, F)}	240.659	9.577	0.007	0.008	10	220.647

The most parsimonious model has the lowest corrected Akaike's information criterion (AICc) value. ΔAICc: difference of AICc value from best suited model (bold); NP: number of parameters; AICc weights, model likelihood and deviance as defined by Cooch and White [44]. Model notations– φ: survival, f: female, m: male, B: nest building, L: laying, I: incubation, N: nestling, F: fledgling, +: given stages are modelled as 1 interval.

Akaike's information criterion corrected for a small sample size AICc [45, 46] was used to identify the most parsimonious model. The model with the lowest AICc was taken as the best representation of the data.

In order to compare various intervals (parts of the breeding season or nest stages), survival rates for the whole interval or season were calculated by raising the DSR of each interval to the power of the number of days within the interval, and the survival rate for the whole season was calculated by multiplying the interval survival rates [44]. General 95% CI were obtained using the same methods (following [47]). All estimates were calculated according to this formula. Hence, all survival values presented in the results section (including those in tables and figures) refer to cumulative survival rates for the whole season, part of a breeding season or a nest stage (as stated in individual sentences/legends), and not DSR.

In order to illustrate the difference in survival rates between our seasonal results and annual estimates obtained by others, we recalculated our estimates from the best model to the length of the whole year using the formula:

$$\Phi y={\Phi s}^{\frac{ny}{ns}}$$

Where *Φy* is a survival estimate for a period of 365 days, *Φs* is the survival estimate for the breeding season obtained from our best model, *ny* is the number of days in the year, and *ns* is the number of days in the breeding season.

## Results

### Survival analysis

A total of 570 individuals (318 females and 252 males) were colour-ringed during the study period, monitored throughout the whole breeding season, and used for analyses.

**Fig 1:**
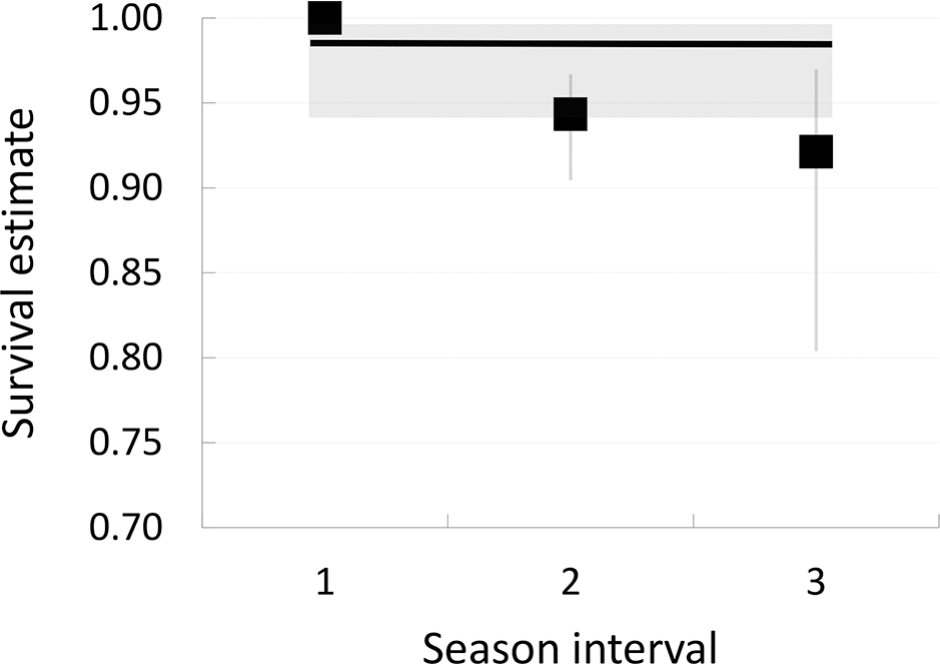
Changes in survival rates of reed warblers throughout the breeding season. Survival rates and 95% CI of female (squares) and male (line) reed warblers were estimated by the best model (Model 1, Table 1). The best model divided the breeding season into three equal intervals for females (1, 2, 3; 27 days each) and was constant for males.

Our best suited model combined splitting the breeding season into 3 equal intervals for females, and maintaining a constant survival rate throughout the season for males (Table 1, model 1). Values for males were very high throughout the whole breeding season (0.985, 95% CI: 0.941–0.996), whereas female survival decreased as the season progressed—values dropped from 1.000 (95% CI: 1.000–1.000) to 0.921 (95% CI: 0.804–0.970); Fig 1. Furthermore, reed warbler survival rates for the whole breeding season calculated from this model showed significant differences between male and female survival (0.985, 95% CI: 0.941–0.996 and 0.869, 95% CI: 0.727–0.937, respectively). Extrapolating the estimates for a period of 365 days (Φ_y_) gave a value of 0.950 (males) and 0.623 (females).

**Fig 2:**
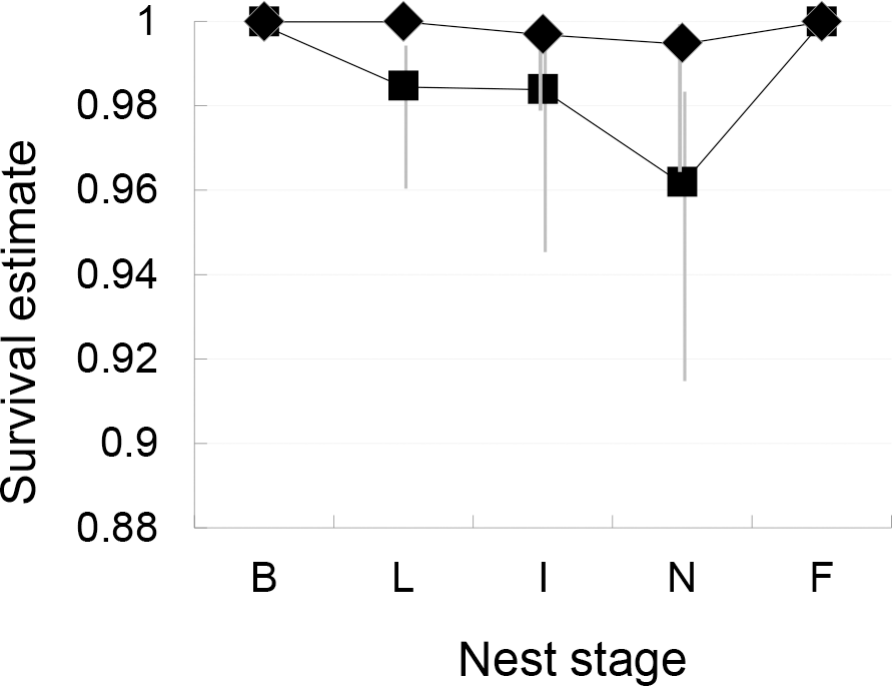
Survival rates of reed warblers at all nest stages. Survival rates and 95% CI of female (squares) and male (diamonds) reed warblers were estimated by the basic model (Model 5, Table 2). Notations–B: nest building, L: laying, I: incubation, N: nestling, F: fledgling. Survival estimates refer to cumulative survival throughout the whole stage (not DSR).

**Fig 3:**
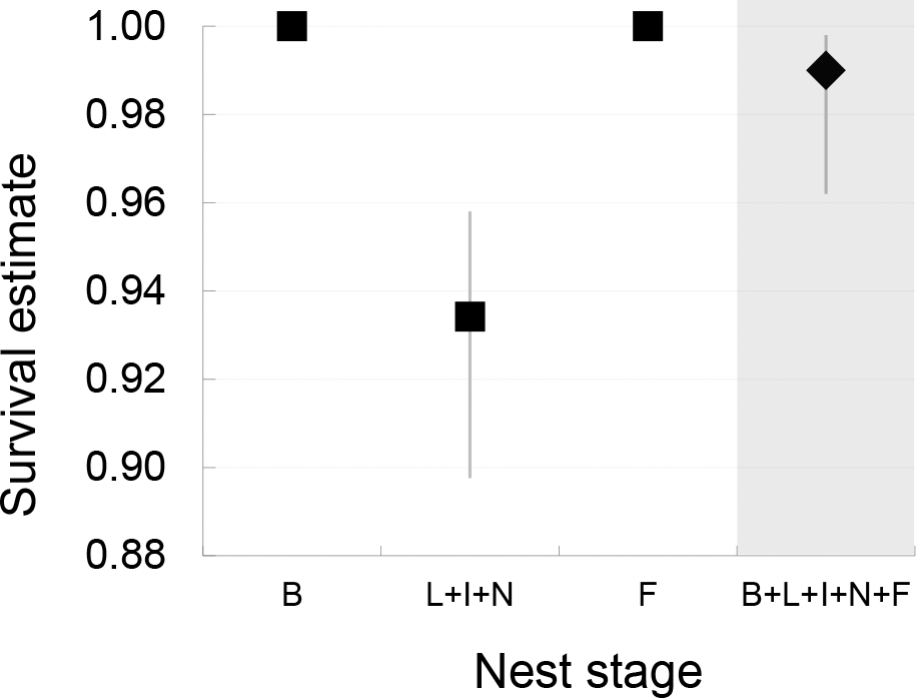
Survival rates of reed warblers for given nest stages estimated by the best suited model. Survival rates and 95% CI of female (squares) and male (diamonds) reed warblers estimated by the best model (Model 1, Table 2). Notations–B: nest building, L: laying, I: incubation, N: nestling, F: fledgling, +: given stages are modeled as 1 interval. Survival estimates refer to cumulative survival throughout the whole stage (not DSR).

All cases of mortality were recorded during the egg-laying, incubation, and nestling periods, with no deaths observed during nest building and feeding of the fledged young. Nesting stages showed to have a significant effect on female survival, but did not affect males. When all stages were considered separately (Table 2, model 5) female survival rates were lowest for the laying (0.985, 95% CI: 0.960–0.994), incubation (0.984, 95% CI: 0.945–0.995), and nestling stage (0.962, 95% CI: 0.915–0.983). Males also exhibited lowest values during the incubation (0.997, 95% CI: 0.979–1.000) and nestling (0.995, 95% CI: 0.964–0.999) stages, but values were much higher than for females (Fig 2). The most supported model (Table 2, model 1) showed that female mortality is the highest and relatively similar during the laying, incubation, and nestling periods (estimates revealed significantly lower survival rates at these stages: 0.934 (95% CI: 0.898–0.958) compared to 1.000 (95% CI: 1.000–1.000) estimates during the building and fledgling stages; Fig 3), while males maintained a similar survival rate throughout various nesting stages (0.990, 95% CI: 0.962–0.998).

## Discussion

Two major natural factors have been proposed to affect avian mortality during the breeding season and nesting cycle: predation of adults, especially on the nest, and mortality related to reproductive effort [1, 22]. Results obtained in this study suggest that both can influence survival of parental reed warblers during the breeding season.

To our knowledge, this study is the first to analyse adult survival for all different stages of the nesting cycle in altricial birds. Our results showed that, in contrast to male survival, which was constant throughout various nesting stages, female survival was affected by the stage of the nesting cycle. Female mortality was highest during the laying, incubation, and nestling stages. The timing of decreased female survival corresponds to increased time spent on the nest by birds in our population [40]. We did not find a single case of mortality during the nest building or fledgling stages, when parental presence on the nest is extremely limited.

Our results showed a significant difference between female and male survival (0.869, 95% CI: 0.727–0.937 and 0.985, 95% CI: 0.941–0.996, respectively) throughout the whole breeding season. Reed warblers exhibit biparental care: both parents are engaged in incubation and feeding of their young, and the frequency of feeding is similar for both sexes or higher for females [35, 48]. However, females spend more time incubating than males, and only females cover the eggs and nestlings at night [40, 48]. Likewise, brooding of the nestlings is provided almost exclusively by females, and occurs for 9 of 11 days of the nestling stage. As a result, time spent on the nest by females (including day and night hours) is much higher than for males. As birds are probably more exposed to predator attacks when on the nest, this could indirectly suggest that the main reason of mortality was on-nest predation (c.f. [19, 22, 31]). Several studies have shown that predation is the main factor limiting adult survival during the breeding season. In particular, cavity nesting birds are frequently killed on nests, most probably because of their limited escape possibilities [22, 32, 34]. However, open-nesters may also suffer high mortality caused by predation (e.g. [19, 30, 31]). This was probably the main reason of mortality of parental birds in our study population, though, as in several other studies [19, 21, 22], we found bird carcasses or their remains in only a few cases.

Decreased adult survival may be a result of cumulative parental investment affecting immunocompetence and other physiological parameters [22, 26]. If this had been the main cause of female deaths, we should expect an increase in mortality throughout the breeding cycle from laying until fledging, but we did not find such a pattern. However, costs related to reproductive effort could have been responsible for a decrease in survival rates with the progression of the breeding season. Despite some overlap in confidence intervals, estimated values for females declined from 1.000 (95% CI: 1.000–1.000) to 0.921 (95% CI: 0.804–0.970), while male survival was constant at 0.985 (95% CI: 0.941–0.996). In our population an increase in adult mortality was observed to correspond with the decline in hemoglobin levels of parental birds as the reproductive season progressed (L. Halupka, unpublished data). As hemoglobin levels are used as a measure of condition, this may suggest that a decline of condition may be a possible factor influencing survival during the breeding season. Although nest predation patterns very enormously in our population from year to year, and a seasonal increase in nest predation was observed only in some seasons [33], this explanation is not mutually exclusive with our suggestion presented above that the most frequent reason of adult mortality is probably predation (c.f. [22, 34]): a decline in condition of parental birds increases predation risk. Furthermore, parental birds in our population increase nest defense intensity with the advancement of the breeding season [49], exposing themselves to higher predation risk [27]. Finally, increased mortality across the breeding season may be a result of a larger proportion of inexperienced first-year breeders nesting in our population as the season progresses. Younger birds may be more prone to predation, and cumulative costs of reproduction may be more severe for them than for experienced breeders. However, we could not properly test this hypothesis, as first-year breeders cannot be distinguished from older individuals in the species [35].

The estimated survival rates of reed warblers during the breeding season were relatively high in comparison with other passerines studied during the same part of the annual cycle: 0.68–0.86 [15, 20, 22, 31], though a few authors reported higher survival at this time in passerine species (0.95–1.00, [2, 19]), and especially in waders (0.99–1.00, [11, 13]). Relatively low mortality rates of adults in our population might be associated with: 1) open nests enabling a much easier escape compared to nest cavities, 2) a high-density habitat where reed warblers maneuver easily [35], which is not always the case for their predators, 3) "noisiness" of the habitat caused by its density and fragility of reed stems, which enables early detection of an approaching predator, 4) vigilance of reed warblers on the nest–in contrast to some other species, they usually leave their nests well before someone approaches. At present it is not possible to determine which of these factors are of key importance, and carefully designed experimental studies are needed to determine this.

Annual survival rates previously recorded for the species 33–60% [50, 51, 52, 53, 54, 55] were low compared to extrapolated results found in this study (0.950 and 0.623 for males and females, respectively), suggesting that much of the species mortality occurs outside the breeding season, during migration or at wintering grounds (see [11, 13, 56]). In contrast to data available for other passerine species (e.g. [13, 19, 22, 29]), the two studies examining sex-specific annual survival for reed warblers have not shown a clear, consistent pattern in differences between male and female survival [52, 55]. Therefore, it is difficult to determine whether higher female reproductive costs could have long-lasting effects and may contribute to their lower annual survival. As reproduction and survival are complex processes, influenced by multiple factors, future research should aim to further explore these topics and experimentally determine the short (during current breeding seasons) and long term (future annual survival) effects of increased individual costs of reproduction on mortality.
